# Fighting Disease by Propaganda

**Published:** 1921-02-05

**Authors:** 


### THE PUBLIC WELL-BEING.

### Fighting Disease by Propaganda.

We are glad to observe the formation of new branches of the National Association for the Prevention of Tuberculosis. This organisation has done, and is still doing, valuable work, chiefly by means of propaganda, and that its task is not nearly so difficult as it was twenty years ago, when the British public were inclined to treat rather contemptuously the efforts of a few far-sighted and public-spirited persons to educate them in the cause of the disease and the means of prevention, is due largely to the unwearying persistence of the Association itself. Even now the value of propaganda is only beginning to be apprehended. It is the very breath of progress. It gives shape to public opinion, without which there would be no Ministry of Health and no public health measures, and certainly no co-operation from the general community in the work of health officials and medical officers.

### Public Prejudice.

By way of illustrating the distance we have travelled in the last twenty years Sir St. Clair Thomson (member of the Council of the Association) mentioned the other day that twenty years ago the Association could not induce any railway company to put up certain notices in their waiting-rooms and passenger carriages because the word "spit" occurred in them! In addition the companies argued that their first-class passengers did not spit upon the floor, and that the third-class passengers would spit on the floor whether notices were put up or not. Now notices containing the objectionable English word are put up and paid for by the companies themselves. The same uninformed squeamishness is evident at the present time in many quarters with regard to venereal disease. It is something gained that the disease can be named in public without members of a mixed audience rising from their seats in fear and disgust and leaving the room. It is still more that lectures on the subject are reported in the newspapers and discussions by correspondence not only tolerated but welcomed. But that public opinion is not yet completely won over to the necessity of dragging this evil thing into the light of day and strangling it with knowledge and candour is proved by an incident which has just occurred at Manchester.

Some time ago the Manchester City Council, on the advice of the Public Health Committee, caused posters to be placed in the underground lavatories of the city intimating that free treatment for venereal disease could be obtained at several Manchester hospitals, and that men who had exposed themselves to infection could be provided with facilities for disinfection at certain specified stations. In this common-sense proceeding the Council has the approval and support of the Ministry of Health. But various social religious bodies are opposed to the provision of facilities for early disinfection on the ostensible ground that its offer of comparative immunity from disease is an incitement to loose living. They have vigorously protested against the wording of the posters, which, they say, neglect the moral side of the question and lay too much stress on the physical side. As far as we can make out, what these people would like is a series of poster-sermons, only delicately hinting at the physical consequences of the disease and avoiding all reference to preventive treatment, in the hope that the public which sees them will not only read them, but be sufficiently impressed to act upon them. Do the religious bodies really imagine that posters of this type will be worth the paper they are printed upon? If they do, their faith in human nature is more spacious than their appreciation of realities.

### The Modern View.

Our view is the attitude of the Council—that the Council is the guardian of the bodily health of the citizens, and that the moral issues are the concern of the organisations which have been protesting. If the section of the public to whom the information about venereal disease is now being supplied in this manner all over the country do not attend to the moralities of the question preached from church pulpits, they are not very likely, one might suppose, to be more impressed by sermons in print published in underground lavatories. The moral question is one; the physical question is another. But of this we may be quite certain: the disease whose appalling ravages on the health of the people are now notorious will not be stamped out by moral preaching. It will only be checked and overcome by public enlightenment as to its physical causes and consequences and as to the methods that must be promptly employed for preventive treatment. It is difficult to understand how any persons with practical experience of the world and with the most elementary knowledge of the disease can remain blind to this simple fact.

